# Experiences and lessons learned from a patient‐engagement service established by a national research consortium in the U.S. Veterans Health Administration

**DOI:** 10.1002/lrh2.10421

**Published:** 2024-04-16

**Authors:** Tracy L. Sides, Agnes C. Jensen, Malloree M. Argust, Erin C. Amundson, Gay R. Thomas, Rebecca Keller, Mallory Mahaffey, Erin E. Krebs

**Affiliations:** ^1^ Center for Care Delivery and Outcomes Research, Minneapolis Veterans Affairs (VA) Health Care System Minneapolis Minnesota USA; ^2^ U.S. Military Veteran Venice Florida USA; ^3^ GR Thomas Advisors, LLC Fort Myers Florida USA; ^4^ U.S. Military Veteran Red Wing Minnesota USA; ^5^ VA Pain/Opioid Consortium of Research Veteran Engagement Panel Minneapolis Minnesota USA; ^6^ School of Medicine, University of Minnesota Minneapolis Minnesota USA

**Keywords:** engagement infrastructure, patient engagement, patient‐centered research, research networks, Veterans

## Abstract

**Introduction:**

Meaningful engagement of patients in the research process has increased over the past 20 years. Few accounts are available of engagement infrastructure and processes used by large research organizations. The Pain/Opioid Consortium of Research (Consortium) is a U.S. Department of Veterans Affairs (VA) research network that provides infrastructure to accelerate health research and implementation of evidence‐based health care. The Consortium's key activities include facilitating Veteran‐engaged research and building community between Veterans and VA researchers. This report sought to describe experiences and lessons learned from the first 3 years of a national research engagement service, featuring a Veteran Engagement (VE) Panel, established by the Consortium.

**Methods:**

We gathered authors' experiences to describe development and operation of the Consortium's VE Panel. Engagement staff collected program evaluation data about partners (Veterans and researchers), projects about which the VE Panel consulted, and meeting attendance during operation of the engagement service.

**Results:**

We created a 12‐member VE Panel; all of whom had lived experience with chronic pain, prescription opioid medication use, or opioid use disorder. Engagement staff and VE Panel members implemented an engagement service operational model designed to continuously learn and adapt. The panel consulted on 48 projects spanning the research process. Seventy‐eight percent of panel members, on average, attended each monthly meeting. VE Panel members and participating researchers reported high satisfaction with the quality, ease, and outcomes of their engagement service experiences.

**Conclusions:**

This work provides an illustrative example of how a national research consortium facilitated Veteran‐engaged research and built community between Veterans and VA researchers by developing and operating an ongoing engagement consulting service, featuring a VE Panel. The service, designed as a learning community, relied on skilled engagement staff to cultivate high quality experiences and outcomes for all partners.

## 1 INTRODUCTION

Meaningful engagement of patients and caregivers in research is a critical component of learning health systems.^1^, ^2^ Over the past 20 years, momentum to engage patients as partners in the health research process has grown.^3^, ^4^, ^5^ Beyond a moral imperative to involve patients in identification of research priorities and conduct of research about them,^6^ meaningful patient engagement can improve the feasibility, acceptability, rigor, and relevance of health research and positively influence the experience of researchers and patient partners.^7^, ^8^, ^9^, ^10^, ^11^

Funders have encouraged or required patient engagement in health research.^12^, ^13^ In the United States., the Patient‐Centered Outcomes Research Institute (PCORI),^14^ requires patient engagement throughout studies,^7^ manages a research portfolio on patient and stakeholder engagement,^15^ and offers training and resources.^16^ Research institutions such as the U.S. National Institutes of Health (NIH), and Department of Veterans Affairs (VA) Health Systems Research (HSR), within the Office of Research and Development, have also implemented programs and practices to incorporate the voices of patients across the research process.^17^, ^18^, ^19^

As the largest integrated learning health care system in the United States, VA funds research on health issues that affect Veterans.^20^ Increasingly, VA HSR leadership seeks to include Veterans in all steps of the research cycle.^20^ In 2015, VA HSR began supporting Veteran engagement in research by establishing a national Veteran Engagement (VE) Workgroup to explore approaches and develop recommendations for promoting engagement.^21^ HSR intramural funding now requires consideration of Veteran engagement in research proposals.^22^ These initiatives have increased Veterans' involvement in agenda setting, study design, methods, interpretation, and dissemination of results.^9^, ^10^, ^23^, ^24^, ^25^, ^26^

Sharing of models and lessons learned may support successful implementation of patient engagement efforts in VA and beyond; however, few accounts are available of engagement infrastructure and processes used by large research organizations.^19^, ^27^, ^28^

This article describes experiences and lessons learned from the first 3 years of an engagement service established by a national VA research consortium to facilitate Veteran‐engaged research and build community among Veterans and VA researchers. We describe the service's context, activities, output, and evaluation results. We also share templates and tools to adapt and use in other settings to promote meaningful patient engagement.

## 2 FRAMEWORK AND METHODS

**FIGURE 1:**
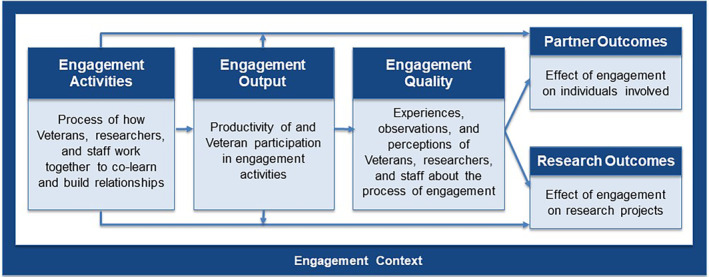
VA Pain/Opioid Consortium's engagement service framework.

We adapted the PCORI engagement in research theory of action model^29^, ^30^ to the circumstances of the Consortium's engagement service (Figure 1) to use as a framework to describe our experiences. We use the term “engagement service” to reflect the Consortium infrastructure and processes involved with creating and operating a standing engagement panel as a resource available to researchers. The PCORI model frames engagement activities, their quality, and the resulting research and partner outcomes within a given context made up of resources and circumstances that may influence how engagement occurs and its impact. We added an *output* domain to capture concepts of productivity and participation, adjusted descriptions to a VA setting, and included only shorter‐term research outcomes to align with our scope of evaluation.

We employed multiple methods of data collection including authors' experiences, surveys, and interviews (Table 1). Our approach, in keeping with the principle of partnership in research engagement, involved staff, Veterans, and researchers to characterize the nature and outcomes of the Consortium's engagement service. Authors TS, AJ, EA, MA, and MM are engagement service staff, GT is a stakeholder engagement consultant, EK is a physician‐researcher, and RK is a VE Panel member.

**TABLE 1:** Engagement service framework domains, concepts, and data sources.

Domain	Concept	Description	Data source
Engagement context		Resources and circumstances surrounding the practice of engagement in research that may affect how engagement occurs and its impact	Authors' experiences
Engagement activities	Development	Approaches to planning and creating the Veteran Engagement (VE) Panel	Authors' experiences and VE Panel records
	Operation	Approaches to recruitment and scheduling of researchers and steps in the VE Panel meeting process	Authors' experiences
	Continuous Improvement	Processes for monitoring VE Panel principles and practices, tracking output, evaluating process and outcomes, and applying knowledge gained to adapt the engagement service	Authors' experiences
Engagement output	Productivity	Amount, types, and topics of engagement consultations provided by VE Panel over a specified time period	VE Panel tracking spreadsheet
	Participation	VE Panel member attendance over a specified time period	VE Panel attendance records
Engagement quality	Engagement experience	Veterans' and researchers' experience of engagement and its underlying principles, as well as satisfaction with engagement structure, processes, and outcomes	*For Veterans*: Post‐meeting online surveys Periodic group and 1:1 discussions *For Researchers*: Post‐meeting online surveys Interviews ~6 months after meeting
	Partnership functioning and group dynamics	Veterans' perception of value contributed to research, psychological safety, and power sharing during the engagement process	
	Equity and inclusiveness	Cultural, social, demographic, and economic inclusiveness of engagement and the degree to which Veterans' perspectives are validated and valued	
	Trust between partners	Level of trust between Veterans, staff, and researchers while working together	
Partner outcomes	Veterans	Effect of engagement experience on individual Veterans who participate as members of the VE Panel	
	Researchers	Effect of engagement experience on individual researchers who consult with the VE Panel	
Research outcomes		Effect of Veteran engagement on research projects	

### 2.1 Engagement context

#### 2.1.1 VA research setting

In 2022, investigators at 104 VA medical centers across the country conducted research spanning the translational spectrum, with a total research budget of $2.3 billion.^31^ VA HSR funds collaborative research to identify, evaluate, and rapidly implement evidence‐based strategies that improve care quality and safety.^32^ HSR intramural funding requires consideration of Veteran engagement in research proposals.^22^ Although the full scope of Veteran engagement activities and practices across VA Research has not been reported, engagement infrastructure to facilitate Veteran involvement in research is documented in individual studies,^33^, ^34^ a research resource center for engaging rural Veterans,^35^ and at HSR's 20 research centers.^9^, ^10^ Investigators face common barriers of time, funding, and training to meaningfully involve Veterans in the research process.^36^, ^37^

#### 2.1.2 About the Pain/Opioid Consortium of Research Veteran Engagement Panel

The Pain/Opioid Consortium of Research (Consortium) is one of four HSR research networks created in 2019 to provide infrastructure to accelerate health services research and implementation of evidence‐based health care. The Pain/Opioid Consortium is led by three co‐Principal Investigators (including EK) and a Consortium Leadership Committee comprising VA researchers affiliated with eight geographically dispersed VA medical centers. The Consortium's engagement service facilitates Veteran‐engaged research and builds relationships of mutual respect between Veterans, staff, and VA investigators doing research related to pain, opioid prescribing, or opioid use disorder. To achieve these purposes, an engagement team created a standing VE Panel available to consult with VA pain/opioid researchers with intended outcomes of: (1) co‐learning and satisfaction among all partner groups (Researchers, Veterans, and Staff) and (2) change(s) to a researcher's project attributable to consultation with the Consortium's engagement service.

### 2.2 Engagement activities

#### 2.2.1 Engagement service development

Past experiences of EK, AJ, EA, and MM establishing VE panels for large, multi‐year research studies coupled with engagement best practices^3^, ^4^, ^38^, ^39^, ^40^ informed the development of the engagement service operational model, which features a standing VE Panel that relies on skilled staff to (1) facilitate interactions between Veterans and researchers, (2) develop effective agendas and facilitation guides, and (3) maintain relationships with Veterans between engagement consultations. The Consortium engagement service staff includes part‐time effort from five VA staff (including one Veteran) and one contracted consultant, totaling approximately 1.0 full‐time equivalent.

#### 2.2.2 Engagement service planning

Staff embedded guiding stakeholder engagement principles of partnership, respect, flexibility, and equity throughout the planning, creation, and operation of the engagement service. To depict how this was operationalized, authors conferred to (1) designate, from among commonly cited guiding principles of engagement,^4^, ^27^, ^41^, ^42^ *partnership*, *respect*, *flexibility*, and *equity* as those most aligned with the values that drove the planning, creation, and operation of the VE Panel, and (2) generate examples reflecting each principle in practice (Table 2).

**TABLE 2:** Examples of guiding principles reflected in engagement service activities.

Activity area	Guiding principle			
	Partnership	Respect	Flexibility	Equity
Planning	Veterans explicitly named as “partners” by Consortium Engagement staff recognized as partners in engagement service	Panel members compensated for their time Panel members' experiences and outcomes included in evaluation plan	Built learning cycles into engagement service plans Designed panel meeting process for flexibility, enabling staff to swap between meeting roles, as needed	Diverse panel membership named as an explicit goal Mechanisms to manage power differences embedded into operational model
Creation	Panel members described as partners in recruitment materials Orientation activities designed to foster bonds both among panel members and with engagement staff	Panel application process uncomplicated and brief Panel applicant interview structured and conducted as a two‐way discussion	Original in‐person orientation adapted to virtual meeting platform Per panel preference, members have 2‐year renewable terms and no specific attendance metric (attend as often as able)	Panel recruitment spanned U.S. and emphasized diverse backgrounds Panel orientation structured to highlight value of Veterans' lived‐experience expertise
Operation	Engagement staff oriented visiting researchers, during meeting planning process, to role of panel members as research *partners* rather than *subjects* or *patients* Engagement staff dedicated at least one panel meeting per year to review process and outcome evaluation results and consider next steps Engagement staff requested monthly feedback/ recommendations from panel members to refine meeting processes	Engagement staff and visiting researchers developed key facilitation questions for each VE Panel meeting Engagement staff regularly shared back to panel how Veterans' feedback made a difference to visiting researchers and their projects	Engagement staff/visiting researchers varied methods for meeting structure (breakout sessions, polling, large group discussion) to best answer key facilitation questions Engagement staff arranged an extra meeting with only the female panel members for two consultations focused on women Veterans Engagement staff modified post‐meeting evaluation survey for VE Panel members after first year of operation in response to member feedback	Visiting researchers came from an array of disciplines (medicine, psychology, pharmacy, nursing) and varied career stages (post‐doctoral to senior VA leadership) Engagement staff intentionally structured and facilitated panel meetings to invite and value perspectives of all participants Participation in extra consultation opportunities tracked by engagement staff to ensure fair distribution among VE Panel members

*Note*: “Creation” includes VE Panel recruitment, selection, orientation. “Operation” includes researcher recruitment and scheduling; engagement panel meeting process (plan, prepare, conduct, closeout and summarize); monitor, evaluate, and adapt activities.

#### 2.2.3 Engagement panel creation

##### Recruitment and selection

Recruitment for a Veteran panel took place January to May 2020 with goals that every panel member had lived experience relevant to chronic pain, prescribed opioid use for pain, and/or opioid use disorder and that, collectively, members reflected the diverse Veteran population. Prospective members were recruited to work with researchers as compensated consultants ($50/hour), not as research subjects. We recruited by disseminating recruitment materials (Appendix S1) through (1) the Consortium's Leadership Committee; (2) existing VA research engagement panel liaisons across the country; (3) national groups of female Veterans and Veterans in recovery; and (4) university Veteran liaisons.

**FIGURE 2:**
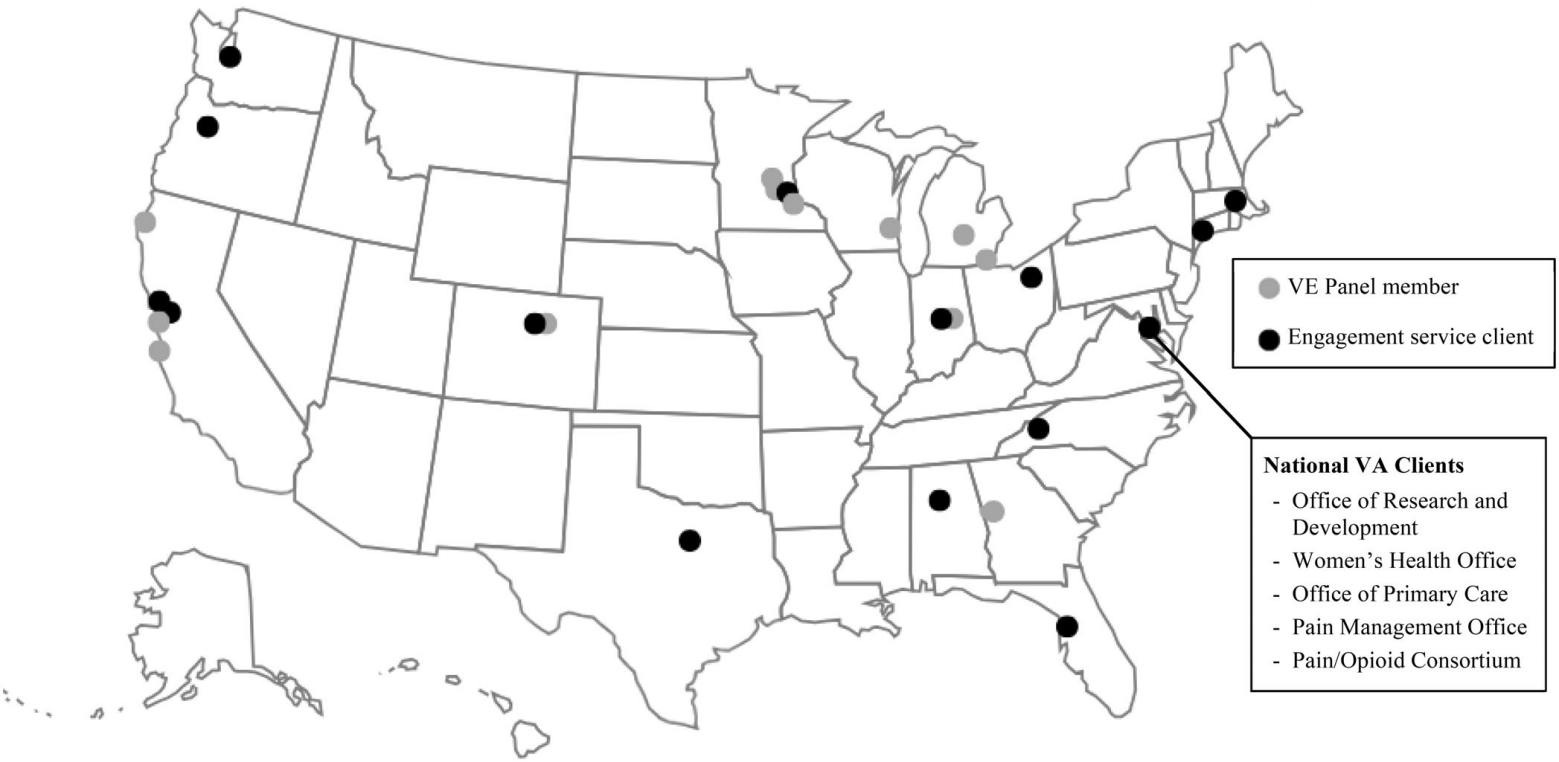
Residential locations of Veteran Engagement (VE) Panel members and VA locations of engagement service clients. Engagement service VA client locations are defined by the scope of the consult (i.e., national or local) and, for local clients, the city and state of the VA medical center of the lead researcher.

Recruitment materials instructed prospective panel members to contact staff. Panel applicants (n = 15) completed a brief questionnaire about their background and interest in the panel. Staff conducted a 30‐min telephone interview (Appendix S2) with each applicant to discuss Consortium priorities, expectations of panel members, communication styles, and comfort with technology (eg, virtual meeting platforms). One staff member conducted and took notes on all interviews and then consulted with a second staff member to produce a synopsis. Consortium PIs selected 12 candidates and all 12 accepted positions. Members' self‐reported characteristics are depicted in Table 3 and Figure 2. Two Veterans resigned from the VE Panel in 2022 and were replaced by new members in March 2023.

**TABLE 3:** Characteristics of Veteran engagement panel members^a^ (N = 12).

	No. (%)
Sex	
Male	5 (42%)
Female	7 (58%)
Race^b^	
White	5 (42%)
Black/African American	6 (50%)
Asian	1 (8%)
Native Hawaiian/Other Pacific Islander	2 (17%)
Ethnicity	
Hispanic or Latino/Latina	1 (8%)
U.S. Military Branch	
Army	6 (50%)
Navy	3 (25%)
Air Force	2 (17%)
Marine Corps	1 (8%)
Service Era^b^	
Vietnam (1961–1975)	1 (8%)
Post‐Vietnam (1975–1990)	7 (58%)
Persian Gulf War (1990–1991)	5 (42%)
Gulf War (1991–2001)	7 (58%)
Post‐9/11 (2001‐Present)	6 (50%)
Current life role^b^	
Employed for pay (full or part‐time)	7 (58%)
Retired	4 (33%)
Student	2 (17%)
Caregiver or Parent^c^	5 (42%)
Volunteer Worker	7 (58%)
Residence, Rural/Urban	
Urban/Suburban	10 (83%)
Rural	2 (17%)

^a^ Self‐described characteristics provided by current panel members, August 2023.

^b^ Members may have one or more racial identities, served during one or more service eras, and have one or more life roles. As a result, numbers sum to greater than 12 and percentages to greater than 100% for these three characteristics.

^c^ Category includes being a parent, guardian, caregiver to an adult or child, or any combination of these roles.

##### Panel member orientation

An orientation was created to develop strong group bonds, a deep understanding of the VE Panel member role and responsibilities, and confidence in members' capacity to meaningfully contribute to group activities. Part 1 (1 h) featured introductions, technology review, virtual meeting tips, confidentiality and membership agreements, and panel member payment processes. Part 2 (2 h) included group icebreaker activities; overview of research, the Consortium, and how input would be used by researchers; a discussion of privacy and confidentiality expectations; and a post‐meeting evaluation. Part 3 (2 h) included additional relationship‐building activities, review of member feedback from the previous meeting, and the first consulting session in which the panel provided feedback on recruitment materials for a study. Orientation exercises emphasized that the unique value of member feedback is derived from their lived experience and opinions, not knowledge of research methods or processes. At the request of VE Panel members, staff established a consistent meeting schedule to allow members to plan around other commitments.

#### 2.2.4 Engagement service operation

**FIGURE 3:**
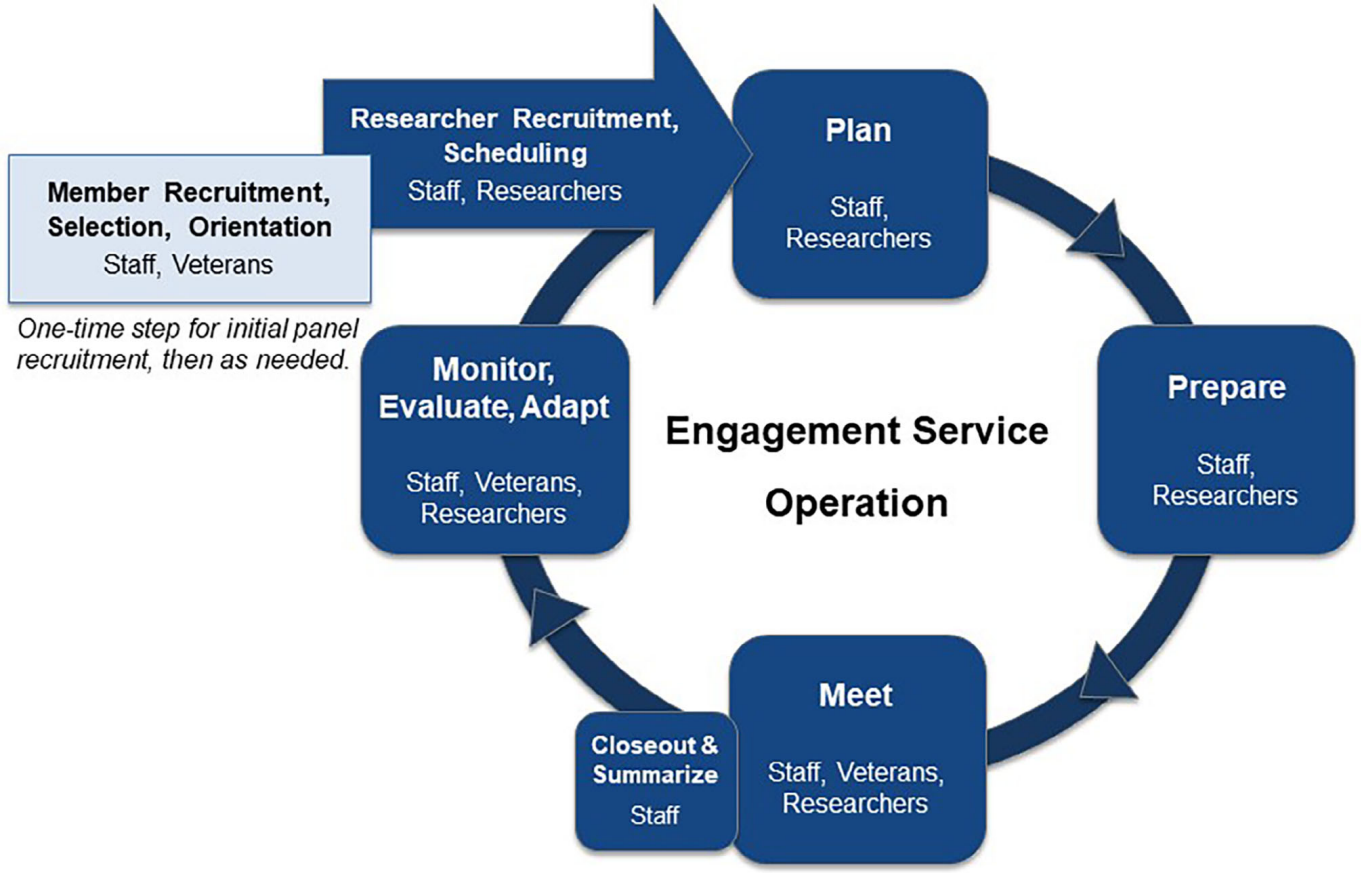
VA Pain/Opioid Consortium's engagement service operational model.

Beginning August 2020, staff and panel members provided active engagement services to VA researchers. Staff facilitated three phases of operations: researcher recruitment and scheduling; meeting processes; and continuous improvement (Figure 3).

#### 2.2.5 Researcher recruitment and scheduling

To fill monthly VE Panel meeting slots in the first year, Consortium staff actively marketed the engagement service by email, announcements, and targeted outreach to VA pain/opioid researchers. After 1 year, word‐of‐mouth and routine promotion in the Consortium's newsletter were sufficient to recruit researcher clients to the engagement service. Interested VA researchers contacted the Consortium's VE staff. To decrease time spent explaining the VE Panel meeting process, staff developed a one‐page overview of the meeting process to share with prospective researchers (Appendix S3).

#### 2.2.6 Engagement panel meeting process

Each cycle of a VE Panel meeting involved four phases: (1) plan, (2) prepare, (3) meet, and (4) closeout and summarize. Three roles (facilitator, co‐facilitator/technology monitor, and notetaker) with predefined duties allowed staff to rotate among roles.

1. *Plan*. Two months before a scheduled VE Panel meeting, a staff member solicited client researcher completion of an intake form about their project and goals for the consultation (Appendix S4) and scheduled a 45‐min planning session. During the session, two meeting facilitators oriented the researcher(s) to the Panel's meeting process and identified aspects of their project that could most benefit from Veteran input.
2. *Prepare*. Before each VE Panel meeting, a lead facilitator drafted a facilitation guide, which included a plain language summary of the researcher's study to share with the Panel and open‐ended key questions and probes to elicit members' perspectives. The co‐facilitator and engagement consultant reviewed and edited draft documents. The lead facilitator and engagement consultant conducted a practice meeting with the researcher(s) several weeks before the VE Panel meeting. This live‐editing practice session enabled adjustments to better manage time, improve process, or reprioritize key questions.

After the practice session, the lead facilitator made final edits to the meeting materials. Staff mailed hard copies of meeting materials to Panel members 2 weeks in advance and sent electronic copies via email 1 week prior to the meeting. Several days before the meeting, staff sent an email reminder, including meeting materials, to all attendees. Hard copy meeting materials lessened panel members' visual burden by allowing them to focus on virtual attendees rather than meeting material handouts on‐screen.

3 *Meet*. During the 2‐h VE Panel meeting, the lead and co‐facilitator took turns leading the discussion and managing comments provided via the meeting platform's chat function. Meetings were not recorded. A staff notetaker captured Veteran feedback, any electronic poll results, and the chat transcript. The researcher's sole responsibility was to be fully present to listening to and interacting with the VE Panel members, including asking or answering clarifying questions.

Meetings started with an icebreaker question to help foster connections among panel members, staff, and the researcher(s). Icebreakers helped make the virtual meetings feel more personal, gave all meeting participants a chance to speak right away, and provided a transition to the discussion topics.

Next, facilitators briefly recapped feedback provided by members at the previous VE Panel meeting and how the researchers planned to use it (Appendix S5). This “reporting back” is a best practice in stakeholder engagement, clearly communicating the value of members' input and demonstrating engagement principles of partnership and respect.

The main portion of the meeting began with a facilitator sharing the plain language overview of the researcher's project followed by the facilitators leading discussions based on the two to four key questions developed with the researcher. To support discussion, meetings sometimes included features such as visual aids, demonstrations, virtual breakout sessions, and live poll questions. Early in the VE Panel's first year, members indicated a preferred approach for eliciting their feedback during meetings: invite two to three members to verbally respond to each key question rather than a “round‐robin” or volunteer format. This provided two benefits: (1) a few members could provide in‐depth responses and (2) space was created for less outgoing members to actively participate. This practice was augmented by inviting all other members to also offer their input by adding comments or questions via the meeting platform's chat function. One facilitator monitored the chat function and incorporated specific comments into the meeting dialogue. Staff tracked and rotated panel member speaking order for each discussion to support equitable opportunities to contribute from meeting to meeting.

4 *Closeout and summarize*. Immediately following each meeting, payments to panel members were processed. Typically, within 1 week after each meeting, staff summarized Veteran feedback shared during meeting discussions, appended a copy of relevant chat comments, and sent these to the researcher(s).

#### 2.2.7 Continuous improvement

We established engagement service practices to monitor VE Panel processes, track output, evaluate process and outcomes, and continually apply the knowledge gained.

##### Monitor

After each VE Panel meeting, staff spent up to 15 min reflecting on outcomes of the meeting and any concerns with group member agreements, guiding principles, or pace. If warranted, reflections were triaged for either immediate follow‐up or consideration at a quarterly staff reflection meeting.

##### Engagement service tracking

Staff captured characteristics of each VE Panel meeting, researcher, and project in a tracking spreadsheet with a “dashboard” of calculated fields to track engagement service productivity and patterns. We reviewed final VE Panel meeting facilitation guides to verify VE Panel consultation category (i.e., study, governance, implementation), topic (e.g., research priorities, study design), type of study (e.g., clinical trial), and study stage (e.g., study design, recruitment). Other characteristics tracked in the spreadsheet included Veteran attendance and speaking order, researcher location, project title, and funding status (Appendix S6).

##### Post‐meeting evaluation

We collected short‐term process and outcome evaluation data via online surveys of Veterans and researchers. For Veterans, we developed a panel member survey based on an existing HSR Veteran Engagement Toolkit.^43^ Questions assessing experience with effectiveness, ease of participation, and sense of partnership in the engagement consultation were emailed immediately following each meeting (Appendix S7). After the VE Panel's first year, based on Veteran feedback, we changed our short‐term evaluation process to an open‐ended virtual poll question at the conclusion of each meeting (“Please share feedback on today's meeting. What went well, and what is one thing you would like to see improved?”). We also collected qualitative process and outcome evaluation data from Veterans, at least annually, during a VE Panel meeting with facilitated discussions dedicated to group maintenance and planning, with optional one‐on‐one conversations after the meeting with a staff member.

To assess experiences of researchers who used the engagement service, we used a survey previously developed by the Minneapolis VA Center for Care Delivery and Outcomes Research Veteran Engagement Core to solicit short‐term satisfaction with effectiveness, ease of participation, sense of partnership, learning, and changes in perception or project activities from the engagement consultation (Appendix S8). We also collected follow‐up process and outcome evaluation data from researchers approximately 6 months after each VE Panel meeting using semi‐structured interviews. During each interview, staff captured notes that were reviewed by a second staff member to corroborate identified outcomes. Questions focused on (1) changes resulting from their consultation with the VE Panel, (2) recommendations of the Consortium's engagement service to colleagues, and (3) free‐form comments about experiences with the engagement process (Appendix S9).

To close feedback loops, staff reported short‐term researcher evaluation results at the next month's VE Panel meeting. Twice per year, staff batched 6‐month follow‐up researcher process and outcome evaluation results and reported them at a VE Panel meeting.

##### Adapt

We established routine learning cycles, which were opportunities for staff and Veterans to reflect on evaluation data and experiences and to adapt engagement service operations to new learning or circumstances. Staff met quarterly to reflect on monitoring, tracking, and evaluation data; changing VE Panel circumstances; and their own experiences. Staff presented recommended operational changes to VE Panel members for consideration prior to implementing them (e.g., altering meeting processes, broadening engagement services to new lines of work, opportunities to build community around VA research). Beginning in 2021, ad hoc consultations in addition to regular VE Panel meetings were scheduled on a case‐by‐case basis with a subset of VE Panel members. Staff tailored engagement planning and preparation activities for these ad hoc consultations according to individual circumstances.

#### 2.2.8 Templates and tools

The appendices contain templates and tools others can adapt and use to create and operate an ongoing engagement service for a research network or enterprise. Table 4 lists materials included.

**TABLE 4:** List of appendices.

Activity area	No.	Appendix title
Creation	1.	Veteran Engagement (VE) Panel member description and recruitment flyer
	2.	VE Panel applicant interview questions
Operation	3.	Overview of VE Panel meeting process for researchers
	4.	Researcher intake form
	5.	Example template to recap how researchers plan to use VE Panel feedback
	6.	VE Panel consultation characteristics routinely tracked
	7.	Veteran post‐meeting evaluation surveys (versions used in Year 1 and Year 2 to present)
	8.	Researcher post‐meeting evaluation survey
	9.	Researcher 6‐month follow‐up interview questions

### 2.3 Data analysis

Descriptive statistics were used to summarize data from the VE Panel tracking spreadsheet, attendance records, and structured evaluation surveys. Two staff members used content analysis^44^ of project titles to summarize research topic areas covered in VE Panel consultations. All open‐ended process and outcome evaluation data were categorized by two staff members into domains of engagement quality, partner outcomes, and research outcomes, as defined in Table 1.

## 3 RESULTS

### 3.1 Engagement output: productivity and participation

Between September 2020 and August 2023, the Consortium's VE Panel consulted on 48 different projects with VA clients (Figure 2) on topics spanning the research process from research priority‐setting to implementation of study findings (Table 5). The 48 engagement consultations occurred via 36 VE Panel meetings and 10 ad hoc consultations (e.g., email review of recruitment material, Veteran presentation to research community). Research topics included a variety of pain and opioid use interventions and health services (Table 6). Between September 2020 and August 2023, 78% of VE Panel members, on average, attended each meeting. Attendance decreased the second year, and new members were recruited to replace two members who resigned due to changes in life circumstances. Attendance increased the third year (Table 7).

**TABLE 5:** Characteristics of Veteran Engagement (VE) Panel Consultations,^a^ Sep. 2020 to Aug. 2023.

All consultations (n = 48)	
Consultation category and topic	No. (%)
Governance	7 (15%)
Strategic or operational planning for Consortium or VE Panel	6
VA research priorities for pain and opioid use conditions	1
Study^b^	31 (64%)
Study design	25
Recruitment	14
Data collection	3
Analysis or interpretation	3
Dissemination	7
Implementation	10 (21%)
Presentation for VA research or clinical community^c^	3
Research communication product for a Veteran audience	6
Clinical practice guidelines	1

Study consultations by study type (n = 31)	
Study or project type	No. (%)
Feasibility study	5 (16%)
Observational study	3 (10%)
Clinical trial	11 (35%)
Quality improvement or evaluation project	7 (23%)
Secondary data analysis	2 (6%)
Other (e.g., systematic review, standard measure instrument)	3 (10%)

^a^ Includes VE Panel meeting consultations (n = 38 across 36 meetings) and other consultations (n = 10).

^b^ Study consultations are inclusive of quality improvement or program evaluation projects and may involve one or more study stages as topics.

^c^ Veteran presentation venues included in‐person, virtual, and recorded.

**TABLE 6:** Research topics of engagement service consultations.

Topic category	Example project title
Pain management	Feasibility and Acceptability of Music Imagery, and Listening Interventions for Analgesia A Mixed Methods Feasibility Study of Integrated Tele‐Yoga Therapy for Midlife and Older Women Veterans with Chronic Pain Options for Pain management and Tapering of Opioids using Nonpharmacological Strategies Implementation Facilitation of Screening, Brief Intervention, and Referral to Treatment for Pain Management for Veterans Separating from Military Service Peers Enhancing Engagement for Pain Services Feasibility, Pain and Functional Outcomes of a Novel Mixed Reality‐Based System to Manage Phantom Pain for Patients with Lower Limb Amputation: A Pilot Study
Opioid use disorder (OUD)	Predictors, Processes and Outcomes of Buprenorphine Discontinuation Factors that Impact Long‐Term Buprenorphine Continuation and Discontinuation among Older Veterans with OUD: a mixed‐methods study Using Medical Record Technology to Help Identify Veteran Unmet Social Needs, with Goals of Reducing Opioid Overdose and Engaging More Veterans in Addiction Treatment
Pain and mental health or substance use disorders	Understanding Care Experiences of Veterans with Pain and/or Addiction Who Use VA Homeless Patient Aligned Care Teams Evaluation of a Computer‐Based Chronic Pain Treatment for Veterans with OUD Engaged in Telemedicine for Buprenorphine Moving to Improve Pain and Depression in Older Adults Study Learning How to Adapt Shared Medical Appointments for Pain to Better Fit the Challenges Faced by Veterans with Trauma

**TABLE 7:** Veteran Engagement (VE) Panel Meeting Attendance^a^ from Sep. 2020 to Aug. 2023.

		Veteran attendees per meeting			Average attendance per meeting
		5 to 7	8 to 10	11 to 12	
	Operational Year	No.	No.	No.	
2020‐2021 (11 meetings)		0	6	5	10.6
2021‐2022 (11 meetings)		1	10	0	8.5
2022‐2023 (12 meetings)		3	6	3	9.0
All years (34 meetings)		4	22	8	9.4

^a^ Excludes two VE Panel meetings convened for only female Veterans.

### 3.2 Engagement quality and outcomes

VE Panel members reported high satisfaction and meaning from their contributions to research that occurred during engagement consultations (Table 8). Seventy‐one total responses across post‐meeting surveys during the VE Panel's first 12 months of operation reflected high satisfaction related to questions about effectiveness, ease of participation, sense of partnership, and return on investment of Veterans' time in the engagement consultation (average 3.9–4.0 on 4.0 scale). Veterans specifically identified that staff's routine reporting on how Veterans' input was used by researchers and strong relationships among VE panel members and staff contributed to their high satisfaction with the engagement experience.

**TABLE 8:** Engagement quality and outcomes.

Evaluation area	General examples of quality or change	Specific examples or changes attributable to Veteran engagement service
Engagement quality		
Engagement experience	Satisfaction with processes and outcomes Ease of participation Co‐learning Sense of partnership	“The whole [meeting] process was so incredibly well‐thought out and organized. The prep with staff before the meeting made the actual time with the [VE Panel] a truly enjoyable experience… I could really focus on being present to the conversation with the Veterans. The meeting was such a great success in terms of the amount and variety of feedback we received that directly influenced our project.” – Researcher “During the pre‐meeting prep, engagement staff really helped me shape our questions into a productive structure and get to the points I wanted to get to.” – Researcher “I loved the use of the poll to kickstart the discussion… it was interesting to see our commonalities and divergences regarding the poll question.” – Veteran “Continue doing what you're doing. The meetings run smooth.” – Veteran
Partnership functioning and group dynamics	Mutual valuing of time invested in VE Panel meeting process Perceived respect, psychological safety, and power sharing	“I just thought it was really well‐done, by both the facilitators and the panel, which was informed, engaged, articulate, and enthusiastic.” – Researcher “I felt like a human the whole time. I was neither a disrespected nor elevated human. I got the sense that we were all equals.” – Researcher “Keep up the great work … I love being a part of this panel!” – Veteran
Equity and inclusiveness	Sociodemographic and life‐experience diversity of VE Panel membership Perception that perspectives of all partners are valid and valued	“I appreciated the diversity of age, race, gender, and life experiences—that is one of the biggest strengths of this panel.” – Researcher “Facilitator did a superb job in moderating the inputs, ensuring each Veteran had time for input, and redirecting lengthy or tangential inputs with sensitivity.” – Veteran “Everyone has a voice and is listened to” – Veteran
Trust between Veterans, staff, and researchers	Perceived openness in sharing among partners during meetings Perceived quality of rapport between Veterans and engagement staff	“We really, really loved the facilitators… the panel was comfortable with them, and they were so in tune.” – Researcher “I appreciate how comfortable it is to share personal experiences about sensitive topics during meetings.” – Veteran
Veteran outcomes	Sense of serving fellow Veterans by contributing to VA research Increased self‐advocacy as patients of the VA health care system Deeper understanding of scope, purpose, and value of VA research New relationships and professional opportunities	“The [VE Panel] has absolutely changed my life. I'm proud to be part of a great team influencing VA research. I really feel like I'm making a difference for my fellow Veterans.” – Veteran “My time as a [VE Panel] member has taught me to better collaborate with my VA healthcare providers and more fully address my medical concerns.” – Veteran “[VE Panel] participation has increased my knowledge, and grateful appreciation, of the breadth and depth of VA health research wholly in support of Veterans” – Veteran “[This new professional connection] is a dream come true for me, and it opens doors that I previously thought were out of reach… the support of the VE Panel and engagement staff has instilled in me a renewed sense of purpose and determination and I am immensely grateful for that.” – Veteran
Researcher outcomes	Increased motivation to engage Veterans as partners in future research Increased self‐efficacy around engaging Veterans in the research process Initiated other Veteran engagement activities unrelated to project of consult	“The meeting preparation and conversation with the VE Panel helped my study and me. I feel better prepared to work with and consider Veteran perspectives in my research. I've already started applying this learning in my other projects.” – Researcher “The time with the VE Panel was such a personally rewarding experience, in terms of now feeling so excited and optimistic that I can actually do research in a way that incorporates the perspectives of the Veterans, the patients, who my work is intended to benefit.” – Researcher “Because of our experience with the [Consortium's VE Panel] and process, we've advocated for more and earlier Veteran engagement in other priority‐setting processes across VHA.” – Researcher
Research outcomes	Study design, conduct, or translation more Veteran‐centered Increased study recruitment Research proposal more Veteran‐centered	“VE Panel feedback taught us important differences about patients' healthcare experiences of diagnosis and treatment of chronic pain vs substance use disorder that have changed our analysis approach.” – Researcher “Implementing the [VE Panel's] suggestions led to the improvement we've seen in recruitment for our study” – Researcher “We integrated comments from the [VE Panel], sometimes verbatim… and changed the protocol… reviewers of this [successful] application loved that we met with the VE Panel in preparation for our submission.” – Researcher

Abbreviations: VA, U.S. Department of Veterans Affairs; VE, Veteran engagement; VHA, Veterans Health Administration.

Researchers reported high satisfaction in short‐term evaluation surveys with the quality, ease, and outcomes of their experiences with the engagement service (26 of 30 researchers responded, average 3.8–4.0 on 4.0 scale). Researchers consistently reported increased self‐efficacy and motivation to engage Veterans in their current and future research because of their experience with the VE Panel, staff, and the meeting preparation process (Table 8). Additionally, in post‐meeting evaluations, all researchers identified specific changes to their projects they attributed to their consultation with the VE Panel (Table 8).

Based on researcher and VE Panel member evaluations, critical skills and functions that staff contributed include communication among all parties, group facilitation, translation of study questions and overviews into plain language, and serving as “power brokers” to support a “level playing field” for collegial interactions between researchers and Veterans.

## 4 DISCUSSION

We presented the experience of planning, creating, operating, and evolving a standing VE Panel over a 3‐year period as a Veteran engagement service offered by a national VA pain/opioid research consortium. Results provide evidence that such engagement infrastructure, intentionally developed and maintained as a learning community, can produce Veteran‐inspired changes to research processes, and deliver positive engagement experiences and effective, meaningful outcomes for researchers, Veterans, and staff.

Engagement partners (Veterans, researchers, or staff) emphasized keys to successful experiences and results for partners as: panel member recruitment and compensation, panel orientation, relationship development and maintenance, facilitators addressing power dynamics, and monitoring and evaluating engagement quality and outcomes. These observations are consistent with others' findings in the context of research studies,^12^, ^45^, ^46^, ^47^ local research centers,^10^, ^23^ and research networks.^27^ Our experience suggests that these elements also contributed to another measure of success for ongoing engagement panels: sustained participation by panel members over time.

Participating researchers' reflections indicate that participation in the Consortium's VE Panel meeting process acted as a form of experiential learning to enhance their capacity to meaningfully engage Veterans in future research. Other teams that also rely on skilled staff reported similar findings.^10^, ^28^, ^48^ Our experience adds to the growing body of evidence^10^, ^48^ that durable patient engagement infrastructure can provide researcher capacity‐building benefits in addition to addressing time and resource constraints, commonly reported by researchers as barriers to meaningful patient engagement in research processes.^36^, ^47^, ^49^, ^50^

We conceptualized the Consortium's engagement service as a learning community of VE Panel members, staff, and researchers. This framing led to the development of repeatable, yet flexible, engagement processes to operate, continuously generate performance data, reflect on knowledge gained, and adjust practices, as needed, to better achieve the engagement service's goals of facilitating Veteran‐engaged research and building relationships of mutual respect between Veterans, staff, and VA researchers. Functioning in this way, the engagement service could naturally contribute to learning cycles of others within the VA integrated learning health system, as demonstrated by researchers' application of knowledge gained from VE Panel consultations to their studies, quality improvement projects, and beyond. These results underscore the value that standing patient engagement panels, which foster trusting relationships between diverse stakeholders, can add as components in learning health systems.^2^, ^20^, ^51^

The Consortium's engagement service augments existing Veteran engagement activities documented in VA research studies, local research center, and research network contexts (^9^, ^25^, ^34^). VA‐level data about the types, cost, quality, and outcomes of engagement activities and practices are not yet available to gauge enterprise progress toward broad implementation of Veteran engagement throughout the research process. Some evidence exists that large research organizations can move toward capturing and learning from such data; for example, Cope and colleagues cataloged types of engagement infrastructure, approaches, and promising practices among PCORI research networks.^27^

Future research in patient engagement practice should explore funding models of effective stakeholder engagement infrastructure, identify how this infrastructure could best be deployed as part of a multifaceted strategy to advance effective patient engagement across a research enterprise, and solidify a framework of engagement measurement domains and concepts.

Our report has limitations in that it provides the experience of one national effort to advance Veteran engagement in research. We aimed to develop a practical model tailored to the VA integrated health care system, Veteran partners, and a particular range of research areas (pain and opioids), and these processes or templates from our experience may need to be adapted for use in other settings.

## 5 CONCLUSION

A national VA pain/opioid research consortium sought to facilitate Veteran‐engaged research and build community by planning, creating, and operating an ongoing research engagement service, featuring a VE panel, which evolved to provide consultations beyond individual studies to the entire research process. The service, designed as a learning community, relied on skilled engagement staff and consistently produced high quality experiences and outcomes for all partners.

## FUNDING INFORMATION

Supported by Project Award Number COR‐19‐489 from the U.S. Department of Veterans Affairs, Veterans Health Administration, Health Systems Research.

## CONFLICT OF INTEREST STATEMENT

The authors report no potential or actual conflicts of interest regarding this project.

## ETHICS STATEMENT

This activity was deemed an infrastructure project by the Minneapolis VA Health Care System Research & Development Committee.

## Supporting information


**Appendix S1.** Veteran Engagement Panel member description and recruitment flyer.

**Appendix S2.** Veteran Engagement Panel applicant interview questions.

**Appendix S3.** Overview of Veteran Engagement Panel meeting process for researchers.

**Appendix S4.** Researcher intake form.

**Appendix S5.** Template example to recap how researchers plan to use Veteran Engagement Panel feedback.

**Appendix S6.** Veteran Engagement (VE) Panel consultation characteristics routinely tracked.

**Appendix S7.** Veteran post‐meeting evaluation surveys.

**Appendix S8.** Researcher post‐meeting evaluation survey.

**Appendix S9.** Researcher 6‐month follow‐up interview questions.
