# Supplementary material for: Experiences and lessons learned from a patient‐engagement service established by a national research consortium in the U.S. Veterans Health Administration
Source: Learn Health Syst. 2024 Apr 16;8(3):e10421. doi: 10.1002/lrh2.10421 (PMC11257060; doi:10.1002/lrh2.10421)

**Appendix 1: Veteran Engagement Panel member description and recruitment flyer.**

**VA Pain/Opioid CORE Seeks Patient Partners**

**Position Title**: Patient Partner for a national group of VA researchers – the Pain/Opioid Consortium of Research (CORE)

**Position Objectives:**

1. Provide guidance and feedback on studies related to chronic/persistent pain, opioid pain medications, and opioid addiction
2. Support sharing of research results by:

- Identifying ways to spread health information from research studies
- Reviewing products and materials so they are they are useful to patients

**Position Summary**: A Patient Partner is a Veteran who brings unique experience as a health care consumer to the study of chronic pain, opioid pain medication and opioid addiction at the VA. He or she engages as a stakeholder with VA researchers during all phases of research. Patient Partners will include Veterans from across the country.

**Training**: A one-day in-person orientation session with ongoing training as appropriate.

**Duties and Responsibilities**:

- Share perspectives during meetings.
- Prepare for meetings by reviewing documents and agendas.
- Encourage all partners to share ideas and viewpoints.
- Respect Veteran and research confidentiality at all times.
- Commit to attending 2 in-person meetings at the Minneapolis VA. The first one will be in June 2020. Commit to monthly 1- to 2-hour meetings by teleconference for 4 years.
- Use email to send and receive documents.

**Successful Patient Partner Panel members are able to:**

- Share perspectives, insights, and information in constructive ways
- Listen well and respect differing opinions
- Speak comfortably in a group
- Maintain open and productive communication with VA researchers

**Compensation:** $50/hour

**Selection Process:**

- Interested Veterans will be asked to complete a brief statement of interest.
- Applications will be reviewed for personal fit and to create a balanced and diverse panel.
- Selected applicants will be invited to complete a telephone interview.
- Invitations to join the panel will sent out in March 2020.

**If Interested:** Contact [Name] by phone [Phone Number] or email [Email Address].


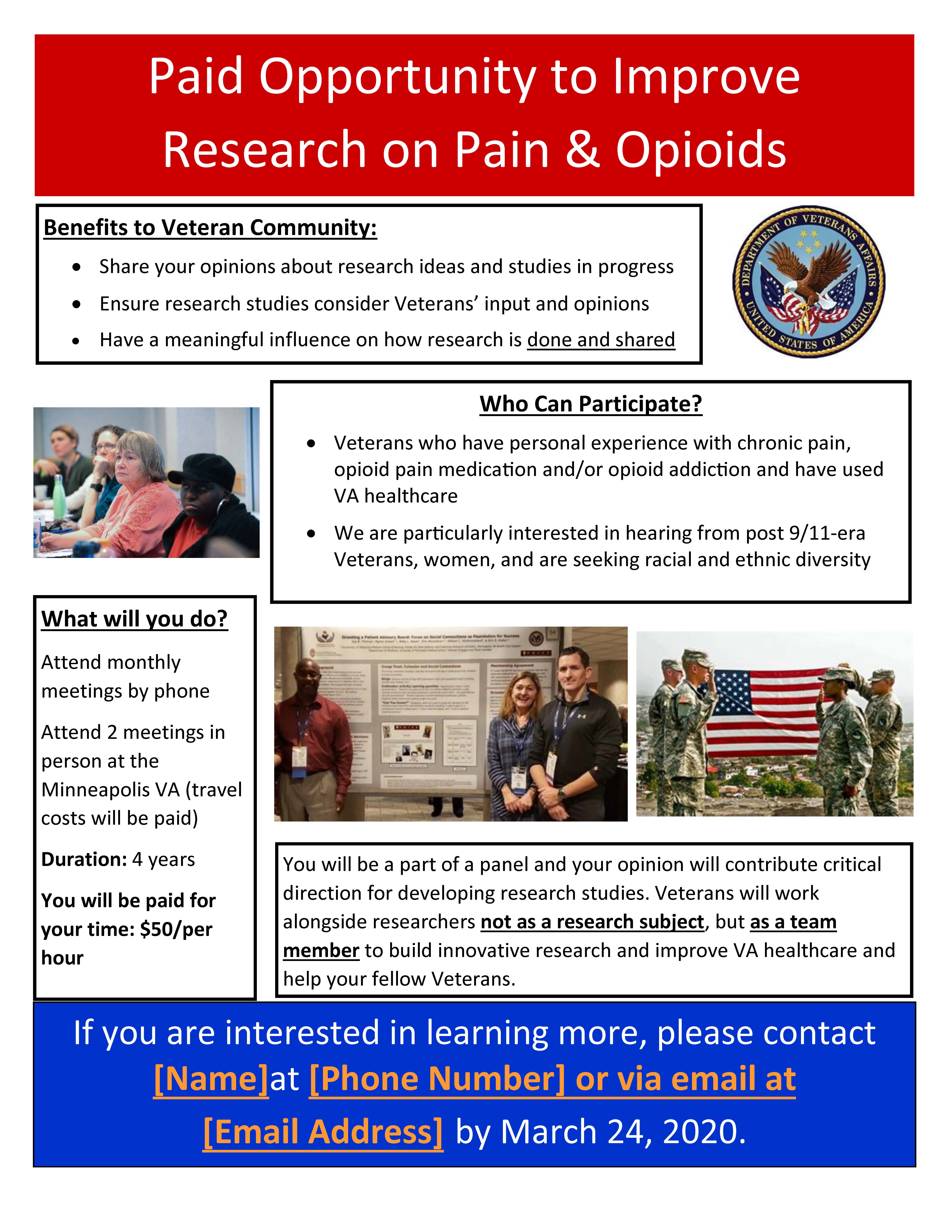

Supplement: Supplementary file 1 — Appendix S1. Veteran Engagement Panel member description and recruitment flyer. [file LRH2-8-e10421-s007.doc]
