# Supplementary material for: Experiences and lessons learned from a patient‐engagement service established by a national research consortium in the U.S. Veterans Health Administration
Source: Learn Health Syst. 2024 Apr 16;8(3):e10421. doi: 10.1002/lrh2.10421 (PMC11257060; doi:10.1002/lrh2.10421)
Supplement: Supplementary file 2 — Appendix S2. Veteran Engagement Panel applicant interview questions. [file LRH2-8-e10421-s005.docx]

**Appendix 2: Veteran Engagement Panel applicant interview questions.**

**Part 1: Initial telephone screener (used when potential member calls staff to express interest)**

1. *Thank you so much for your interest in the Veteran Engagement Panel member position! Let me tell you a little bit more about this opportunity.*

*Our Veteran Engagement Panel meets once a month with researchers from across the country that are working in the fields of chronic pain, opioid medications, and opioid addiction. Members are sent background information prior to that month’s call and then meet to discuss a few key questions that the researcher wants Veteran feedback on to better inform their work. We ask each panel member to commit to a 2-year term, which is renewable.*

- *Your role would be to provide us with feedback on different aspects of our research studies from your personal experience as a Veteran. For example, we might need help figuring out the best ways to recruit Veterans to take part in a particular study. You will be our team member and colleague, NOT a research participant.*
- *We will typically meet up to 12x a year, once a month for two hours. You will be compensated $150 for a 3-hour session, which includes the two-hour meeting plus any pre-work or reading of materials. There are occasionally other opportunities as well which are completely optional.*

Do you have any questions so far?

[If yes]—answer questions; provide explanatory detail.

[If no]—great! I just have a few questions to ask you to see if the panels are a good fit for you. This should take less than 5 minutes.

1. How did you hear about us?
2. Have you used VA health care in the last two years?

[If Yes] Would you be willing to share what types of services you’ve used, or how often you come to the VA?

[If No] Is there a reason you don’t use the VA (i.e., distance, other insurance, etc.?)

1. What made you interested in participating on the panel?
2. Are you comfortable being in a group that includes both male and female Veterans?
3. What years did you serve in the military, and what branch of service?
4. Do you have any questions for me?

**If potential panel member is still interested:** *Thank you for answering these questions! The next steps would be for us to schedule a 15-20 minute-phone interview so that we can get to know each other better. It’s a way for us to learn more about you and an opportunity for you to ask more questions of us about the panel. When would you be available for this phone interview [schedule]? Great! We are set for [time/date]. Can I confirm this is the right number to reach you? We will give you a reminder call for the interview as well* **[Make sure you have all contact info, thank them for their time, and end call].**

**If not interested or unable to participate:** *Thanks for giving us a call. We’re sorry this doesn’t sound like something you are interested in / something you can commit to right now. Please give us a call if your circumstances change.* **[IF RELEVANT]** *May we call you back in the future, if we have more opportunities like this?*

**Part 2: Potential partner interview form**

**VE Staff member fills in this box:**

| **Name:**   **Age:**  **Gender: Race/Ethnicity:**  (Ask age/gender/race/ethnicity directly – if interviewee is willing to share)  **Years served/branch of military:**  **Date of Interview:**  **Interviewer:** |
| --- |

**Introduction:**

*Thank you for your time and interest in the Veteran Engagement Panel. We are looking for people who are interested in working together with researchers to improve research at the VA, specifically around chronic pain, opioid use/suboxone and opioid use disorder. Basically, we want to bring in people like yourself to help researchers better understand what is important to veterans. If you’re selected to be on the panel, you will be expressing your opinions based on your expertise as a veteran. We’ll ask you what you think about research studies we are planning, what you think other veterans might think about our research studies, and how to make our research studies better. We also want to know what you think are the best ways to get the word out to the veteran community about what we find in our research studies.*

*Now that we’ve talked a little about the panel, I’m going to ask you some questions about yourself. Please feel free to share only that which you are comfortable sharing. The interview will take up to 30 minutes.*

**Interview questions:**

1. In brief, please tell us a little about yourself, including your military experience. What years did you serve, and what branch?

2. Our group’s work will focus on three topics: chronic or persistent pain, opioid pain medicines, and opioid addition or suboxone use. We are looking for people with personal life experience in at least one of these so that you could provide input on these issues. Would you be willing to tell us about your experience in these areas?

3. Have you served on a committee at work or in the community? Please share some of your experiences working within a group.

4. **Our panels are not consensus panels, meaning it’s OK if every member doesn’t agree or have the same opinion on a topic.** Have you ever been in a group situation or on a committee when someone had a different opinion than you? What was that like? Was there anything you did to make each other feel comfortable or respected, despite your differences of opinion?

5. Have you ever participated in a research study? Please describe that experience.

6. What interests you about being on a Veteran Engagement Panel?

7. We want to make sure a wide variety of Veteran voices are represented on our panel. If you are willing to share, how would you describe your race or ethnic group? And what is your current age?

8. Can you make a commitment to participate on a monthly video meeting? Are there things in your life that might make it difficult to make this commitment? **Do you have a computer, laptop or iPad with a camera?**

9. Are there barriers such as timing of meetings, caregiving or illness that may need to be accommodated to allow your participation?

10. Finally, what questions do you have for us?

**Part 3: Post-interview notes template (perception of potential panel member)**

1. Overall impression (good fit?/availability):
2. Was the interviewee articulate (provided concise, clear responses)?
3. Did interviewee have ability to reflect (did not go off target or was kept on target if redirected?)
4. Impression of how interviewee would participate in group setting (talk over others? dominate the conversation?).
5. Did interviewee understand panel purpose?
6. Were there any potential concerns with this interviewee?
