# Supplementary material for: Experiences and lessons learned from a patient‐engagement service established by a national research consortium in the U.S. Veterans Health Administration
Source: Learn Health Syst. 2024 Apr 16;8(3):e10421. doi: 10.1002/lrh2.10421 (PMC11257060; doi:10.1002/lrh2.10421)
Supplement: Supplementary file 3 — Appendix S3. Overview of Veteran Engagement Panel meeting process for researchers. [file LRH2-8-e10421-s009.docx]

**Appendix 3:** Overview of Veteran Engagement Panel meeting process for researchers.

**Veteran Engagement Panel Meeting Process Overview**

| **CORE Veteran Engagement Staff** | **Research team** |
| --- | --- |
| ***Before Meeting***   1. Send intake form to Researcher 2. Facilitate VEP planning meetings to understand Researcher priorities 3. Create both a tailored VEP meeting agenda and background materials to help VEP members prepare for meeting discussions 4. Handle all meeting logistics:    1. Confirm meeting time    2. Send VEP the agenda and background materials 1 – 2 weeks before meeting    3. Send out Webex link and reminder to all attendees prior to meeting | ***Before Meeting****   1. 6 weeks before VEP meeting    1. Submit VEP intake form    2. Attend 45-minute VEP planning meeting with CORE VE staff 2. 2 – 3 weeks before VEP meeting    1. Meet with CORE VE staff to run through meeting, finalize materials, and address any questions   ** Structured preparation for the meeting enables us to facilitate an effective VEP meeting for you.* |
| ***During Meeting***   1. Facilitate meeting 2. Take notes on Veteran feedback; copy the “chat” transcript and any poll results | ***During Meeting***   1. Briefly introduce yourself 2. Respond to clarifying questions 3. Be fully present and interact with the VEP |
| ***After Meeting***   1. Pay VEP stipends 2. Send meeting summary to Researcher    1. Notes summarizing feedback    2. “Chat” transcript from meeting    3. Link to online researcher evaluation form 3. About 6 months after VEP meeting    1. Contact you for a brief call regarding your experience with the VEP | ***After Meeting***   1. Complete an online evaluation* 2. About 6 months after VEP meeting   Share how you used feedback in your research plans during a brief phone call with VE staff*  * Optional. |

*For more information, contact: [Email Address]*
