# Supplementary material for: Experiences and lessons learned from a patient‐engagement service established by a national research consortium in the U.S. Veterans Health Administration
Source: Learn Health Syst. 2024 Apr 16;8(3):e10421. doi: 10.1002/lrh2.10421 (PMC11257060; doi:10.1002/lrh2.10421)
Supplement: Supplementary file 4 — Appendix S4. Researcher intake form. [file LRH2-8-e10421-s006.docx]

**Appendix 4: Researcher intake form.**

**Pain/Opioid Consortium of Research (CORE) Veteran Engagement Panel (VEP) ^[[1]](#footnote-2)^**

**Pre-Meeting Planning Tool for Guest Researchers**

*Thank you for your interest in working with our Veteran Engagement Panel (VEP)!*

*Our VEP consists of 12 Veterans from across the country with personal experience with
1) chronic pain; 2) prescribed opioid use for pain; 3) opioid addiction; or a combination of these three.*

*VEP members are acting in the role of paid partners, not research subjects. In that role, they may draw from personal experiences or share anecdotes, but they are representing the experience of many Veteran patients. Our panel includes both men and women from nine different states; some do not currently use VA healthcare. The panel is racially diverse and includes ages 30-60+.*

*Preparation for the VEP meeting will be facilitated by Pain/Opioid CORE staff and an engagement consultant. After seeing your responses below and talking to you about how the VEP can assist with your project, we will help you identify key takeaways the panel members may be able to provide and will write a facilitation guide for the meeting.*

*At the VEP meeting, you will be asked to give a short introduction and to respond to questions from VEP members as they arise, but your role will be as a participant – the Pain/Opioid CORE staff will facilitate the meeting so you can fully engage with the VEP and listen to their feedback.*

*Our staff will take notes during the meeting (provided to you afterwards) and will ask you to complete a brief post-meeting evaluation.*

*To better understand your needs, please complete the brief questionnaire below and return to [Name] at [Email Address].*

___________________________________________________________________________

1. What is the official name of your project?
2. Please provide a title for your project that most people would understand (Plain English).
3. Who is in charge of this project? (Who is the lead researcher?)
   1. Name
   2. Institution/Dept.
   3. Email
   4. Telephone number
4. What kind of feedback do you want from the VEP? (please respond to each item)

| **Feedback wanted on:** | **Yes or no?** |
| --- | --- |
| Overall research plan or idea? |  |
| Plan for how to recruit people for your study? |  |
| Recruitment materials (flyers, brochures, etc.)? |  |
| Survey, focus group or interview questions? |  |
| Review of consent form? |  |
| Review of health education information? |  |
| Feasibility/acceptability of methods, from a patient’s perspective? |  |
| Dissemination of research findings? |  |
| Other (please describe): |  |

1. Why are you PERSONALLY interested in this area of research? What makes you passionate about this project?
2. Who do you want to enroll in your study (if applicable)?
   1. Age range:
   2. Race/Ethnicity:
   3. Geographical location:
   4. Other:
3. What will you ask people in your study to do? (Brief description -- limit to 4 key points)
4. Why might people *want* to join your study? (Payment to participants, medical services, educational information, chance to help others, etc.)
5. Which members of your research team will participate in the VEP meeting?
   1. PI only
   2. PI plus other research team members
   3. Research team members only (no PI)
6. How did you hear about our Veteran engagement panel?

1. Appendix note: The VA Pain/Opioid Consortium is often abbreviated within VA as the Pain/Opioid “CORE” (Consortium of Research). Likewise, the Consortium’s Veteran Engagement Panel, in practice, is commonly shortened to “VEP.” [↑](#footnote-ref-2)
