# Supplementary material for: Experiences and lessons learned from a patient‐engagement service established by a national research consortium in the U.S. Veterans Health Administration
Source: Learn Health Syst. 2024 Apr 16;8(3):e10421. doi: 10.1002/lrh2.10421 (PMC11257060; doi:10.1002/lrh2.10421)
Supplement: Supplementary file 5 — Appendix S5. Template example to recap how researchers plan to use Veteran Engagement Panel feedback. [file LRH2-8-e10421-s008.docx]

**7/28/2021 meeting**

**Appendix 5: Template example to recap how researchers plan to use Veteran Engagement Panel feedback.**

| **How the VEP is making a difference!** | | |
| --- | --- | --- |
|  | | |
| **Provide feedback on study of music imagery and music listening interventions for managing pain**  **Guest researchers:**  Maya Story and Matt Bair, Roudebush VA, Indiana | Impressions on how Veterans view the study interventions:   - “Music therapy” is a new concept for many, however all could relate to “using” music to energize or calm themselves - Music for pain management is a logical extension of ways music is already used - Music therapist can play a valuable role in helping a patient use music in a therapeutic way – making a soundtrack for personal care   Approaches to make study appealing to a broad range of Veterans in recruitment materials:   - Put questions on recruitment flyer to catch people’s attention and draw them in - Focus on practical elements of the study, avoid “touchy feely” language; note that music listening is a key activity in both interventions - Emphasize that creative talent and the ability to play music are not required   Recommendations for study procedures:   - Hybrid delivery model preferred for 8-week interventions (first session in-person) - Ensure technology support is provided - Provide study results to all participants - Be clear about expectations of participants - Be prepared to address tinnitus | - The panel affirmed Maya’s belief that there is Veteran interest in working with music therapeutically - Maya and Matt will incorporate feedback into their study recruitment materials - The researchers valued insights shared about intervention delivery; will reconsider current protocol with virtual-only delivery - Pain/Opioid CORE staff will follow up with Maya and Matt in 3-6 months and share any additional feedback or next steps with the VEP! |
