# Supplementary material for: Experiences and lessons learned from a patient‐engagement service established by a national research consortium in the U.S. Veterans Health Administration
Source: Learn Health Syst. 2024 Apr 16;8(3):e10421. doi: 10.1002/lrh2.10421 (PMC11257060; doi:10.1002/lrh2.10421)
Supplement: Supplementary file 6 — Appendix S6. Veteran Engagement (VE) Panel consultation characteristics routinely tracked. [file LRH2-8-e10421-s004.docx]

| **Appendix 6: Veteran Engagement (VE) Panel consultation characteristics routinely tracked** | | | | |
| --- | --- | --- | --- | --- |
| **#** | **Field Name** | **Description** | **Format** | **Values** |
| 1 | Meeting # | Sequential VE Panel meeting number | Number | Integer |
| 2 | Consultation # | Sequential VE Panel consultation (> 1 consultation may occur at a meeting; consultations may also occur outside of a meeting) | Number | Integer |
| 3 | Meeting Date | Date of meeting or consultation (if outside of a meeting) | Date | Alphanumeric |
| 4 | Consultation Category | Type of consultation provided (one category per consultation) | Text | Drop down list options: - Governance - Research or Project - Implementation (not focused on a single project) |
| 5 | Consultation Topic | General topic or form of consultation provided (one topic per consultation) | Text | Drop down list options: - Clinical Practice Guidelines - Communication Product - Research Priorities - Recorded Presentation - Veteran Presentation - CORE Planning - VE Panel Planning - Other |
| 6 | Researcher | Name(s) of researcher/guest | Text | Open text |
| 7 | Consult # | Number of times researcher/guest has consulted with VE Panel on specific project | Number | Integer |
| 8 | VA location | VA location where researcher/guest is stationed | Text | Open text; CORE, VACO, etc. for consultations with national scope |
| 9 | COIN? | Is VA location listed above a research Center of Innovation (COIN)? | Text | Open text - Yes - No - Other |
| 10 | Official Project Title | Official project title provided to engagement staff during intake process | Text | Open text |
| 11 | Project Title | Plain language version of the official project title for use during VE Panel meeting and on any meeting materials | Text | Open text |
| 12 | Funding Phase | Funding phase of research/project at time of consultation | Text | Drop down list options: - Pre-funding - Post-funding - N/A |
| 13 | Funder | Name of funder for project or proposal, when applicable | Text | Open text |
| 14 | RSF | Visiting researcher received a Rapid Start Funding (RSF) Award from the Pain/Opioid Consortium of Research (CORE) | Text | Drop down list options: - Yes - No - N/A |
| **#** | **Field Name** | **Description** | **Format** | **Values** |
| 15 | Project Phase | Phase of the study or project at time of consultation | Text | Drop down list options: - Preparatory Phase - Execution Phase - Translation Phase - N/A |
| 16 | Project Type | Type of study or project | Text | Drop down list options: - Clinical Practice Guidelines - Clinical Trial - Observational Study - Quality Improvement Project - Secondary Data Analysis - Other |
| 17 | Project Stage 1 | Stage of the study or project on which the consultation is focused (left blank, if not applicable) | Text | Drop down list options: - Agenda Setting - Study Design - Recruitment - Data Collection - Data Analysis - Data Interpretation - Dissemination - Implementation - Evaluation |
| 18 | Project Stage 2 | Stage of the study or project on which the consultation is focused (left blank, if not applicable) | Text | Drop down list options: - Agenda Setting - Study Design - Recruitment - Data Collection - Data Analysis - Data Interpretation - Dissemination - Implementation - Evaluation |
| 19 | Project Stage 3 | Stage of the study or project on which the consultation is focused (left blank, if not applicable) | Text | Drop down list options: - Agenda Setting - Study Design - Recruitment - Data Collection - Data Analysis - Data Interpretation - Dissemination - Implementation - Evaluation |
| 20 | Selected Consultation Outcome(s) | Quotes and direct outcomes from consultation as provided by visiting researcher/guest during post-meeting evaluation survey, interview, or unsolicited email communication. | Text | Open text |
| 21 | Attendance | Panel member attendance tracked in VE Panel meeting payment processing spreadsheet. | - | - |
| **#** | **Field Name** | **Description** | **Format** | **Values** |
| 22 | Speaking order | VE Panel member speaking order tracked in meeting planning spreadsheet. | - | - |
