# Supplementary material for: Experiences and lessons learned from a patient‐engagement service established by a national research consortium in the U.S. Veterans Health Administration
Source: Learn Health Syst. 2024 Apr 16;8(3):e10421. doi: 10.1002/lrh2.10421 (PMC11257060; doi:10.1002/lrh2.10421)
Supplement: Supplementary file 7 — Appendix S7. Veteran post‐meeting evaluation surveys. [file LRH2-8-e10421-s002.docx]

**Appendix 7: Veteran post-meeting evaluation surveys.**

We collected short-term process and outcome evaluation data from Veterans following each of the first 12 Veteran Engagement (VE) Panel meetings (Part A: Year 1) before co-creating, with VE Panel members, a new method to gather feedback at the conclusion of each meeting (Section B: Year 2 to Present). VE Panel members sought a simpler, more free-form way to provide post-meeting feedback.

**Part A: Year 1**

**Veteran Post-Meeting Evaluation Survey**(hyperlink to survey emailed to members who participated in VE Panel meeting)

Veteran Evaluation Survey

Please complete the survey below.

Thank you!

**Meeting date:** [open text response]

| **Please tell us whether you agree or disagree with the following statements regarding the Veteran Engagement Meeting:** | | | | |
| --- | --- | --- | --- | --- |
|  | Strongly Agree | Agree | Disagree | Strongly Disagree |
| Scheduling/communications were handled in a timely manner |  |  |  |  |
| Facilitators managed the allotted time in order to address my questions/comments |  |  |  |  |
| I was satisfied with the Veteran Engagement Meeting |  |  |  |  |
| The Veteran Engagement Meeting process was worth my time |  |  |  |  |
| Researcher’s presentation gave me enough information to provide appropriate feedback |  |  |  |  |
| My fellow veterans and I provided feedback that will improve the research project |  |  |  |  |

**The allotted time for the Veteran Engagement Meeting was sufficient: (Select one)**

- Too much time
- Enough time
- Not enough time

**What do you feel was your contribution to the research project? Please check all that apply.**

- Increased researcher understanding of the veteran community
- Increased researcher sensitivity to the veteran community
- Provided feedback on the feasibility of the project
- Provided feedback on the appropriateness of the project
- Ideas on recruiting research participants
- Ideas on how to inform the veteran community about the project
- Ideas on how to use the results of project to benefit the veteran community
- Other

**If other, please specify:** [Open text response]

**Please suggest at least one way the quality of the Veteran Engagement Meeting could be improved in the future:** [Open text response]

**Part B: Year 2 to Present**

**Virtual Poll Question Posted at Conclusion of Meeting**

The following electronic poll question is posted for 10 minutes in the video conference platform at the conclusion of each VE Panel Meeting:

**Please share feedback on today’s meeting. What went well, and what is one thing you would like to see improved?** [Open text response]
