# Supplementary material for: Experiences and lessons learned from a patient‐engagement service established by a national research consortium in the U.S. Veterans Health Administration
Source: Learn Health Syst. 2024 Apr 16;8(3):e10421. doi: 10.1002/lrh2.10421 (PMC11257060; doi:10.1002/lrh2.10421)
Supplement: Supplementary file 8 — Appendix S8. Researcher post‐meeting evaluation survey. [file LRH2-8-e10421-s003.docx]

**Appendix 8: Researcher post-meeting evaluation survey.**

Researcher Evaluation Survey

Please complete the survey below.

Thank you!

Meeting date: [open text response]

|  | Strongly Agree | Agree | Disagree | Strongly Disagree |
| --- | --- | --- | --- | --- |
| Scheduling/communications were handled in a timely manner |  |  |  |  |
| Facilitators managed the allotted time in order to address my questions/comments |  |  |  |  |
| Veterans with relevant experience to my project were present at the meeting |  |  |  |  |
| I was satisfied with the Veteran Engagement Meeting |  |  |  |  |
| The Veteran Engagement Meeting process was worth my time |  |  |  |  |
| The Veteran feedback summarized in the debrief (post-meeting) was provided in a helpful way |  |  |  |  |
| The meeting improved the quality of my project |  |  |  |  |

**The allotted time for the Veteran Engagement Meeting was sufficient: (Select one)**

- Too much time
- Enough time
- Not enough time

**Would you recommend a Veteran Engagement Meeting to a colleague?**

- Yes
- No

**Would you request a Veteran Engagement Meeting in the future?**

- Yes
- No

**Would you request input again from the individuals participating in this meeting?**

- Yes
- No

**Has your perception about the role of veteran stakeholders in research changed as a result of the Veteran Engagement Meeting?**

- Yes
- No

**If yes, please describe how your perception has changed:** [Open text response]

**What do you feel were the Veteran experts' contribution to the research project? Please check all that apply.**

- Increased my understanding of the Veteran community
- Increased my sensitivity to the Veteran community
- Provided feedback on the feasibility of the project
- Provided feedback on the appropriateness of the project
- Ideas on recruiting research participants
- Ideas on how to inform the Veteran community about the project
- Ideas on how to use results of project to benefit the Veteran community
- Other, please specify

**Other, please specify:** [Open text response]

**What, if anything if anything do you plan to change as a result of the feedback you received from the meeting? (check all that apply)**

- **Research question**
- **Research design**
- **Level of veteran community / patient engagement in research activities**
- **Recruitment/retention strategies**
- **Consent process**
- **Data collection**
- **Data interpretation**
- **Dissemination**
- **Change in number of questions (i.e. survey items)**
- **More patient-centered questions**
- **Less technical/medical jargon**
- **More culturally relevant questions**
- **I do not intend to change anything**
- **Other**

**Please describe other:** [Open text response]

**Is there anything you’d like to share from the Veteran Engagement Meeting?** [Open text response]

**Please suggest up to three ways the quality of the Veteran Engagement Panel meeting could be improved in the future:**

1. [Open text response]
2. [Open text response]
3. [Open text response]
