# Supplementary material for: Experiences and lessons learned from a patient‐engagement service established by a national research consortium in the U.S. Veterans Health Administration
Source: Learn Health Syst. 2024 Apr 16;8(3):e10421. doi: 10.1002/lrh2.10421 (PMC11257060; doi:10.1002/lrh2.10421)
Supplement: Supplementary file 9 — Appendix S9. Researcher 6‐month follow‐up interview questions. [file LRH2-8-e10421-s001.docx]

**Appendix 9: Researcher six-month follow-up interview questions.**

Staff collect follow-up process and outcome evaluation data from researchers approximately six months after a Veteran Engagement Panel (VEP)^[[1]](#footnote-1)^ meeting via semi-structured interviews. Staff use the following questions to spark conversation and take notes. An interview typically takes 10 to 15 minutes to complete.

Reminders for starting interview:

- Greet the researcher, introduce yourself if you haven’t met before
- Thank them for taking time to talk with you
- Remind them of the specific date and topic areas of their VEP meeting

*One purpose of the Pain/Opioid CORE is to support patient-centered research -- providing access to a pain/opioid Veteran Engagement Panel is a *free* research resource offered to the VA pain/opioid research community through funding provided by HSR&D. To document the impacts this panel is having, we'd like to know about your experience since the time you visited with the Pain/Opioid CORE VEP. This shouldn't take longer than 10 to 15 minutes.*

1. *After the meeting, you intended to [insert specific intended outcomes] - what happened?*
2. *Were there any unexpected ways that your meeting experience with the VEP influenced you or your project?*
3. *Are there ways that another visit with the VEP could help advance your project?*
4. *Have you recommended consulting with the VEP to others?*
5. *Is there anything else about your experience with the Pain/Opioid CORE VEP you'd like us to know?*

*Thank you so much for your time.*

1. Appendix note: The VA Pain/Opioid Consortium is often abbreviated within VA as the Pain/Opioid “CORE” (Consortium of Research). Likewise, the Consortium’s Veteran Engagement Panel, in practice, is commonly shortened to “VEP” (pronounced *vehp*). [↑](#footnote-ref-1)
